# Biosynthesis, characterization, and evaluation of bioactivities of leaf extract-mediated biocompatible gold nanoparticles from *Alternanthera bettzickiana*

**DOI:** 10.1016/j.btre.2018.e00268

**Published:** 2018-06-22

**Authors:** Nagalingam M., Kalpana V. N., Devi Rajeswari V., Panneerselvam A.

**Affiliations:** a Department of Zoology, Thiruvalluvar University, Serkadu, Vellore – 14, Tamil Nadu, India; b Department of Biomedical sciences, School of Biosciences and Technology, VIT, Vellore – 14, Tamil Nadu, India

**Keywords:** Gold nanoparticles, *A. bettzickiana*, Zebra fish embryo, Antibacterial, SEM

## Abstract

The objective of the study was to synthesize gold nanoparticles (Au NPs) using leaf extract of *Alternanthera bettzickiana (A. bettzickiana*). The biosynthesized Au NPs were characterized using UV–vis spectroscopy, X-ray Diffraction (XRD), Fourier transform infrared spectroscopy (FTIR), Scanning electron microscopy (SEM), Energy dispersive X-ray analysis (EDX), Zeta potential and Transmission electron microscopy (TEM). Morphologically, the Au NPs showed spherical shaped structures. Size distribution of Au NPs calculated using Scherrer’s formula, showed an average size of 80–120 nm. Au NPs were studied for *invitro* anti-bacterial and cytotoxic activities. Au NPs exhibited significant anti-microbial activity against *Bacillu subtilis*, *Staphylococcus aureus*, *Salmonella typhi*, *Pseudomonas aeroginosa*, *Micrococcus luteus*, and *Enterobacter aerogenes* by agar well diffusion method. The cytotoxic effect of the biogenic synthesized Au NPs against A549 human lung cancer cell lines provided a vigorous evidence of anticancer activity of Au NPs. Further, the toxicity study of the green synthesized Au NPs on *Danio rerio* (Zebra fish) embryo was evaluated. This study reports that colloidal Au NPs can be synthesized by simple, non-hazardous methods and that bio-synthesized Au NPs have significant therapeutic properties.

## Highlights

• The gold nanoparticles (AuNPs) were synthesised using leaf extract of *Alternanthera bettzickiana.*
• The Au NPs were characterized using UV-vis, XRD, FTIR, SEM, TEM and Zeta potential.
• A simple, quick and reproducible method for the environmentally friendly synthesis of Au NPs without the need for expensive reducing agents.
• The cytotoxic effect of the green synthesized Au NPs against A549 human lung cancer cell lines provided a vigorous evidence of anticancer activity of Au NPs.
• The toxicity study of the green synthesized Au NPs on *Danio rerio* (Zebra fish) embryo was evaluated

## 1 Introduction

Recently, metallic nanoparticles have received much attention because of their distinctive properties. Nanoparticles have wide applications in the field of biomedicine such as to deliver pharmaceuticals, for diagnostic purposes as well as for the therapeutic approaches because of its small size [1]. Various methods for synthesizing nanoparticles have been developed to formulate such nanoparticles, including chemical, physical and biological methods. The use of plant extract for the synthesis of nanoparticles could be advantageous over other environmentally benign biological processes by eliminating the elaborate process of maintaining cell cultures [2]. The advantage of using plants for the synthesis of nanoparticles is that they are easily available, safe to handle and possess a broad variability of metabolites that may aid in reduction [3]. Gold nanoparticles (Au NPs) exhibit unique properties which are of great interest for drug delivery, cellular imaging, diagnostics and therapeutic agents. A variety of synthetic procedures for the formation of various shapes and sizes of Au NPs have been reported [4]. Synthesis of gold nanoparticles using plant extract is useful not only because of its reduced environmental, but also because it can be used to produce large quantities of nanoparticles. Plant extracts may act both as reducing agents and stabilizing agents in the synthesis of nanoparticles. In view of its simplicity, the use of plant extract for reducing metal salts to nanoparticles has attracted considerable attention within the last few decades [5].

*Alternanthera bettzickiana*, an ornamental flowering plant grown in tropical and sub-tropical regions. The plant has been scientifically proven to consist of primary and secondary metabolites, such as, alkaloids, carbohydrates, saponins, phenols, flavonoids, diterpenes, tannin, terpenoids, steroid, oxalate, anthocyanin, leucoanthocyanin, Xanthoprotein, coumarin, and glycosides [6]. The leaves are used as traditional medicines for gastrointestinal distress, promoting lactation, treat dementia, and provide nourishment The whole parts of the plant is reported to be useful in purifying and nourishing the blood, apart from this it is known to be soft laxative, a galactagogue, antipyretic, has wound healing property and anti-inflammatory property [7,8]. Here, we demonstrate the synthesis of Au NPs with the aqueous extract of *A. bettzickiana* leaves. The biosynthesized Au NPs were characterized using UV–vis spectroscopy, XRD, FTIR, SEM, EDX and TEM. Further, the antibacterial and cytotoxic properties of the Au NPs were investigated. In addition, toxicity level of Au NPs in *Danio rerio* (Zebra fish) was investigated.

## 2 Materials and methods

### 2.1 Collection of plant material

The plant *A. bettzickiana,* collected during the month of July from Vellore District, Tamil Nadu, India. The taxonomic identification was done by Dr. Sudha, Department of Botany, Voorhees College, Vellore and confirmed by Dr. C. Hema, Department of Botany, Arignar Anna Government Arts College for Women, Walajapet, Vellore, India. The voucher specimen was numbered and deposited in the Department of Zoology, Thiruvalluvar University, Vellore, Tamil Nadu, India for future reference.

### 2.2 Preparation of plant extract

Fresh *A. bettzickiana* leaves were collected and thoroughly washed with running tap water. Then, the leaves were washed with double distilled water and shade dried up to 5 days. Dried leaves were powdered by using mechanical grinder. 10 g of the fine leaf powder was mixed with 100 ml of double distilled water. The mixture was boiled at 80 °C for 10 min in boiling water bath and filtered using Whatmann no.1 filter paper. The extract was collected and stored in a refrigerator at 4 °C for further studies [3,4,9].

### 2.3 Biological synthesis of Au NPs

In a typical nanoparticle synthesis procedure, gold solution was prepared by mixing 1 mM (10^−3^ M) of gold chloride (HAuCl_4_) with 20 ml of double distilled water. About 5 ml of leaf extract was added to 20 ml aqueous gold solution which was heated up to 80 °C for 10 min. Resulted mixture became into cherry red in color after heating. This indicated the reduction of gold metallic (Au^+^) ions to gold (Au°) nanoparticles. The mixture was kept dark condition at room temperature to prevent further reduction of gold ions [4,[10], [11], [12]]

### 2.4 Characterization studies

#### 2.4.1 *Ultraviolet-Visible* (UV–vis) *spectrum analysis*

The reduction of gold ions was monitored by using double beam UV–vis spectrophotometer (Lambda 25, Perkin Elmer, Singapore) of the reaction medium in the wavelength range of 450-700 nm with 1000 mm quartz cell. The resolution of the UV–vis spectrophotometer was 1 nm. The UV–vis spectra of resulting solution were recorded. The spectrum is plotted for wavelength on X-axis against absorbance on Y-axis [13].

#### 2.4.2 Fourier transform infra-red spectroscopy

Functional bio-molecules associated with Au NPs were confirmed by FTIR, which is involved in the reduction of gold ions into Au NPs. Biosynthesized Au NPs was centrifuged at 7000 rpm for 15 min and the pellets were washed with distilled water. Then centrifuging and re-dispersing process was repeated to three times. The samples were dried and analyzed at a wave region of 400 – 4000 cm^−1^ [13]

#### 2.4.3 X-Ray diffraction studies

The obtained Au NPs was purified by repeated centrifugation at 15,000 rpm for 15 min. Then the colloidal form of pellet was collected and dried at 100 °C. After heat drying of the purified Au NPs structures and compositions were analyzed by XRD (Panalytical X’Pert Powder X’Celerator Diffractometer). Crystalline nature of the nanoparticles was analyzed at the 2θ ranges of 20-80°. The dried mixture of Au NPs was collected for the determination of crystalline nature of Au NPsby an X‘Pert Pro X-ray diffractometer operated at a voltage of 40 kV and a current of 30 mA with Cu Kα radiation in a θ- 2θ configuration [13].

#### 2.4.4 Energy dispersive X-ray spectroscopy (EDX)

Energy Dispersive X-ray spectroscopy (EDX) analysis for the confirmation of elemental gold was carried out for the detection of elemental gold. The samples were dried at room temperature and then analyzed for samples composed of the synthesized nanoparticles [14].

#### 2.4.5 Transmission electron microscopy (TEM) analysis

In TEM analysis the sample is first sonicated for 10 min and a drop of this Au NPs solution is loaded on carbon-coated copper grid and the solution is allowed to evaporate for 10 min at room temperature than it was analyzed using HITACHI-H- 7650 at an operating voltage of 80 kV. X-ray diffraction is used to determine crystalline structure. This study was made on the powder samples at room temperature 27 °C on a Rigaku X-ray diffractometer (Miniflex, UK) [13].

#### 2.4.6 Scanning electron microscopy analysis

Scanning Electron Microscopic (SEM) analysis was done using Hitachi S-4500 SEM technique. Thin film samples were prepared on a carbon coated copper grid by just dropping a very small amount of the sample on the grid; extra solution was removed using a blotting paper and then the film on the SEM grid was allowed to dry by putting it under a mercury lamp for 5 min [14].

#### 2.4.7 Zeta potential

The Zeta potential of the synthesized nanoparticles was determined by means of Zeta potential analyzer. The measurement of zeta potential is based on the direction and velocity of particles under the influence of known electric field [14].

### 2.5 Bioassay of antibacterial activities

#### 2.5.1 Test microorganisms

Six different pathogenic bacterial strains were used in the current study. *Bacillus subtilis* (MTCC 441), *Staphylococcus aureus* (MTCC 3940), *Micrococcus luteus* (MTCC 106), *Enterobacter aerogenes* (MTCC 111), *Salmonella typhi* (MTCC-734), and *Pseudomonas aeruginosa* (MTCC 841) were obtained from the Department of Microbiology, VIT, Vellore.

#### 2.5.2 Agar well diffusion method

The antibacterial activity of biogenic synthesized Au NPs was tested by the standard agar well diffusion method. About four wells were made using sterile borer (5 mm) under aseptic condition. Different concentration of Au NPs 10 μl, 20 μl, 30 μl, and 40 μl were added to the well and incubated for 24 h at 37 °C. The zone of inhibition was measured using a ruler and expressed in mm. Ciprofloxacin (Himedia, Mumbai, India) is a reference drug used as a control for test organisms [[15], [16], [17]]

#### 2.5.3 Minimum inhibitory concentration (MIC)

The biogenic synthesized Au NPs was solubilized in 1 ml of dimethyl sulfoxide (DMSO) and serially two fold diluted in Muller Hinton broth to obtain a concentration range of 15.6–1000 mg/ml. The broth containing only DMSO *d*iluted in the same way, which did not influence bacterial growth, was included as controls (Ciprofloxacin). The bacterial strains were suspended in sterile physiological Tris buffer (pH 7.4, 0.05 M), homogenized and adjusted to an optical density of 0.05 at 530 nm (equivalent to 1 × 10^6^ CFU/ml). This suspension was used as the inoculums for the test in the agar plates. Bacterial suspensions (100 μl) were inoculated using a micropipette [18,19].

### 2.6 In-vitro cyto-compatibility assays

#### 2.6.1 Lung cell line culture

Normal and Lung cancer (A549) cell lines were obtained from center for research Faculty, Animal Sciences University and Ramachadra University, Chennai, Tamilnadu. The cells were maintained in Minimal Essential Media (MEM) supplemented with 10% Fetal Bovine Serum (FBS), Penicillin (100 U/ml), and Streptomycin (100 U/ml) in a humidified atmosphere of 50 μg/ml CO_2_ at 37 °C.

#### 2.6.2 MTT assay

The cytotoxicity effect of synthesized gold nanoparticles in normal and lung cancer cell line A549 was determined by the MTT assay. Briefly cancer cells were seeded onto 96- well microplates at a density of 1 × 10^4^ cells/100 μL per well were incubated with gold nanoparticles at the concentrations of 1000 to 1.953 μg/mL for 48-hours. The medium was then removed, and 100 μL of MTT solution (0.5 mg/ml MTT in PBS) was added. Then the cells were incubated for 4 h in CO_2_ incubator and the solutions turn into purple colour indicates formation of formazan. The MTT-purple formazan productions were dissolved in 0.1 N isopropanol/hydrochloric acid (HCl) and optical densities of the solutions were measured by absorbance at 570 nm in an ELISA plate reader [20,21]. Cell viability was expressed as the optical density ratio of the treatment to the control (% of control) as described previously.

$$\%\;growth\;inhibition=\;\frac{100-optical\;density\;of\;treated\;cells}{optical\;density\;of\;control\;cells}\;\times 100$$

### 2.7 Microscopic studies

#### 2.7.1 Light microscopic study

Sub cultured flask containing A549 cells without any contamination were observed under inverted light microscope to study the morphological features, which served as control. The medium inside the flask was decanted and the cells were treated with IC_50_ concentration of gold nanoparticles and the flask was incubated for 48 h at 5% CO_2_ and 37 °C. The flask was taken and observed under inverted light microscope. Cells were considered to be apoptotic if it displayed characteristics of cell shrinkage, reduction in cell population, chromatin condensation and nuclei fragmentation [22].

#### 2.7.2 Fluorescent microscopic study

Two culture flasks with fully grown or 90% confluence reached A549 cells were taken, one serving as control and the other for gold nanoparticles treatment. The medium was decanted and treated with gold nanoparticles and incubated for 48 h at 5% CO_2_ and 37 °C. Cells were trypsinised from both control and NPs treated flask and subjected to centrifugation. Pellet of cells were re-suspended in phosphate buffer saline of pH 7.4. 100 μl of this cell suspension was introduced into microscopic slide along with equal mixture of acridine orange and ethidium bromide for staining. The cells were then viewed under fluorescent microscope. The viable cells (green colour) and dead cells (red colour) were identified by differential uptake of these two fluorescent DNA binding dyes [22].

#### 2.7.3 DNA fragmentation study

Fragmentation of chromatin to units of single or multiple nucleosomes that form the nucleosomal DNA ladder in agarose gel is an established hallmark of programmed cell death or apoptosis. Briefly human cancer cell lines (A549) were treated with gold nanoparticles for 48 h. The cells were pellet and washed twice with cold PBS. Cell pallets were incubated in lysis buffer (1 ml) for 30 min at 60 °C. The clear lysates were separated by centrifugation and incubated with RNase (3 μl) for 30 min at 37 °C A mixture of solvents which consisted of phenol, chloroform and isoamyl alcohol was added and vigorously vortexed for a few seconds before centrifugation. This procedure was repeated twice [23,24].

#### 2.7.4 Protein assay

Agilent high sensitivity protein 250 bio-analyzer was used to identify and quantify the proteins isolated from control cancer cells and in cancer cells after treatment with standard drug and gold nanoparticles. Equal quantity of protein and ladder were subjected to labeling with fluorescent dye and was loaded in the wells of the protein chip along with gel mix and destaining solution. The comb was inserted into the bio analyzer and the comb was run. All the procedures were carried out as described under manufacturer’s protocol and the proteins were identified and quantified with the help of standard ladder using bio analyzer. The aqueous layer was transferred into 100% ethanol (1 ml) and kept at 4 °C. The mixture was again centrifuged to discard the supernatant. The remaining pellet was washed with 70% ethanol. The DNA pellet was resuspended in TE buffer (10 mmol/L Tris−HCI, 1 mmol/L EDTA, pH 8.0) prior to loading (10 μl) onto a 1.5% agarose gel containing 0.5 μg/ml ethidium bromide. Electrophoresis was conducted at 50 V for 4 h. DNA fragments were visualized and photographed under UV illumination. DNA marker was used to estimate the size of DNA fragment [25,26].

#### 2.7.5 Nuclear staining

Human cancer cell lines (A549) were plated at a density of 5 × 10^4^ in 6-well plates and were allowed to grow till 70–80% confluence. Then cells were treated with 10, 25 and 50 μg/mL (selected based on the IC_50_ concentration) of plant based gold nanoparticles for 24 h. The culture medium was aspirated from each well and cells were gently rinsed twice with PBS and subsequently treated with 100 μl of dye mixture (1:1) of ethidium bromide and acridine orange) and viewed immediately under fluorescence microscope. The percentage of apoptotic cells was determined by

$$\%\;apoptotic\;cells=\;\frac{total\;No\;of\;apoptotic\;cells}{total\;No\;of\;cells\;counted}\;\times 100$$

Human cancer cell lines (A549) were plated at a density of 5 × 10^4^ in 6- well plates and were allowed to grow till 70–80% confluence. The cells were then treated with 10, 25 and 50 μg/mL μg/mL of plant based gold nanoparticles for 24 h. Culture medium was aspirated from each well and cells were gently rinsed twice with PBS, fixed with methanol: acetic acid for 10 min, and stained with 50 μg/ml Propidium iodide for 20 min. Nuclear morphology of apoptotic cells with condensed/fragmented nuclei was examined by fluorescence microscopy and at least 1 × 10^3^ cells were counted for assessing apoptotic cell death [27,28].

#### 2.7.6 Western blotting

For the detection of apoptosis-inducing proteins, Human cancer cell lines (A549) at a density of 1 × 10^6^ cells in T25 flask were treated with 10, 25 and 50 μg/mL concentrations of gold nanoparticles for 48 h. Cells at the end of each treatment period were harvested, and isolated total proteins, mitochondrial and cytosolic proteins quantification were carried out as described previously [27]. The lysates from each sample were centrifuged at 13,000 ×g for 10 min and the protein concentration in the supernatant was determined with a BCA protein assay kit (Thermo Fisher Scientific Inc. USA). Equal amounts (40 μg) from each sample of protein lysate were run on 10–12% sodium dodecyl sulfate-polyacrylamide gel electrophoresis (SDS-PAGE). The iBotTM Dry Blotting System (Invitrogen) was used to electro transfer to a PVDF membrane and there after the blot was blocked with 5% nonfat dry milk and 0.05% Tween 20 in PBS at pH 7.4 at room temperature for 1 h. After blocking, the membranes were incubated with anti-caspase-3, anti-caspase-9, anti-Bcl-2, anti-Bax, anti-PARP, anti-p ERK (Thr 202), anti p ERK (Tyr 204), anti ERK 1, anti ERK 2 (Santa Cruz Biotechnology, Inc., Santa Cruz, CA, USA), anti-p53 (Abcam, Cambridge, U.K.) antibodies at 4 °C overnight. The membrane was washed three times with TBST to remove the unbound antibodies. These membranes were then incubated with horseradish peroxidase- (HRP) conjugated goat anti-mouse or antirabbit IgG secondary antibodies (Sant Cruz biotechnology) for 2 h at room temperature with gentle shaking. After washing, bands were visualized by Enchanced chemiluminescent HRP substrate (ECL) kit (Amersham Bioscience) according to the manufacturer’s instructions. The relative abundance of each band was quantified using Image *J* software (version 1.43, NIH, USA) for Windows. Blots were reported with β- actin antibody as a loading control [28].

#### 2.7.7 Analysis of cell cycle distribution

Human cancer cell lines (A549) were seeded at a density of 1 × 10^6^cells/well in a 6-well culture dish. Cells incubated for 24 h were treated with IC_50_ concentrations of Au NPs and subsequently harvested after a 48-h exposure. Trypsinized cells were centrifuged at 1500 rpm at 4 °C, and were then resuspended to a density of 1 × 10^6^ cells/mL in 0.1% sodium citrate solution containing 0.05 mg/ml propidium iodide and 0.1% (v:v) Triton X-100. After a 15 min exposure on ice in the dark, cells were filtered through a 41 μm nylon mesh and analyzed by flow cytometry (FACS system, Becton Dickinson); at least 10,000 cells per sample were analyzed. The percentage of cells in G1, S and G2-M phases was analyzed by cell quest software. All results were expressed as mean ± SD for three replications.

#### 2.7.8 Gene expression studies

Expression of apoptosis-related genes, bcl-2, bax, p21, and p53, was studied using reverse transcriptase-PCR (RT-PCR). Briefly 5 × 10^5^ cells seeded in 3 ml total volume in 6-well multi dishes were incubated in the presence of gold nanoparticles (10, 25, 50 μg/mL) for 48 h at 37 °C. The housekeeping genes β- Actin were used as control. At the end of incubation, the cells were rinsed twice with PBS and trypsinized in trypsine-0.02% EDTA mixture. After centrifugation for 5 min at 500 ×g at 4 °C, the supernatant was removed, and the pellet was used for RT-PCR studies. Total RNA was isolated using Total RNA Isolation System (Promega, France). cDNA was generated by Reverse Transcription System (Promega, France). 10 μL of cDNA product was used for PCR reaction as templates. PCR was carried out using the gene-specific upstream and downstream primers. Initial denaturation at 95 °C for 3 min was followed by a PCR cycle of denaturation at 94 °C for 1 min, annealing at 55 °C for 1 min, and extension at 72 °C for 2 min. PCR products were separated on a 1.5% agarose gel and stained with ethidium bromide. β- Actin was used as an internal loading control. The intensity of individual bands was semi-quantitatively assessed using NIH Image.

### 2.8 Toxicity analysis of zebra fish (Danio rerio) embryo model

Zebra fish is the most popular vertebrate model system and found in freshwater lakes, streams and ponds. It is one of the most sensitive organisms used in ecotoxicity tests. Toxicity studies were conducted with the help of different concentrations of gold nanoparticles to determine the toxic effects seen in the zebra fish embryos. Zebra fish embryos were placed in glass petriplates with egg water. Different concentrations of gold nanoparticles serially diluted and added to the petriplates containing zebra fish embryos to ensure a constant concentration in the beakers. They were covered with aluminium foil and shifted in to a shaker. The petri-plates were shaken constantly at 140 rpm throughout the 12-h, 36-h and 48-h exposure time. Shaking was chosen to minimize sedimentation of particles. After 12 h, 36 h and 48 h of exposure the immobilization of the individuals in each petriplates were assessed under stereo microscope. The experiment carried out in triplicates.

### 2.9 Statistical analysis

Data were analyzed by one way analysis of variance (ANOVA) followed by Fischer’s LSD post-hoc test using SPSS 10.0 software (SPSS Inc. Chicago). Statistical significance was considered at p < 0.05.

## 3 Results and discussions

### 3.1 Synthesis of Au NPs using A. bettzickiana leaf extract

**Fig. 1:**
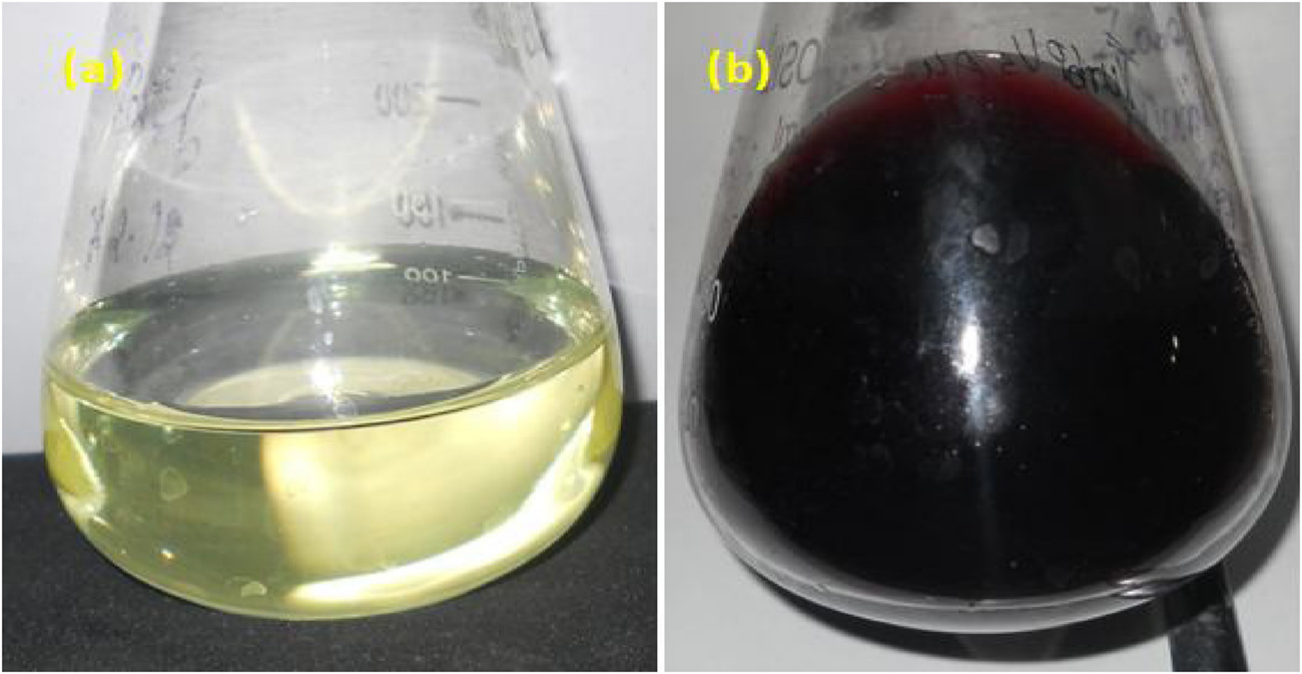
Visual observation of Au NPs formation (a) Yellow colour of the gold ion solution changed into (b) red during the formation of Au NPs.

Synthesis of Au NPs using leaf extract of *A. bettzickiana* was preliminarily confirmed by color change from colorless into cherry red color. The formation of red color (Fig. 1) is a characteristic for the Au NPs [29,30]. The gold ions from the precursor show yellow in color which turns into red color indicates that the gold ions turns into neutral gold atoms. This red color indicates the formation of stable Au NPs [31]. The color change was occurred within 10 min while heating process. After that which remains unchanged indicates the formation of stable Au NPs. The intensity of color is dependent on the time duration so that, nanoparticles formation is directly proportional to time. The result color change obtained in this investigation by is very interesting in terms of identification of potential plants for synthesizing the nanoparticles [32].

### 3.2 Characterization of Au NPs

#### 3.2.1 UV-vis spectrophotometer analysis

**Fig. 2:**
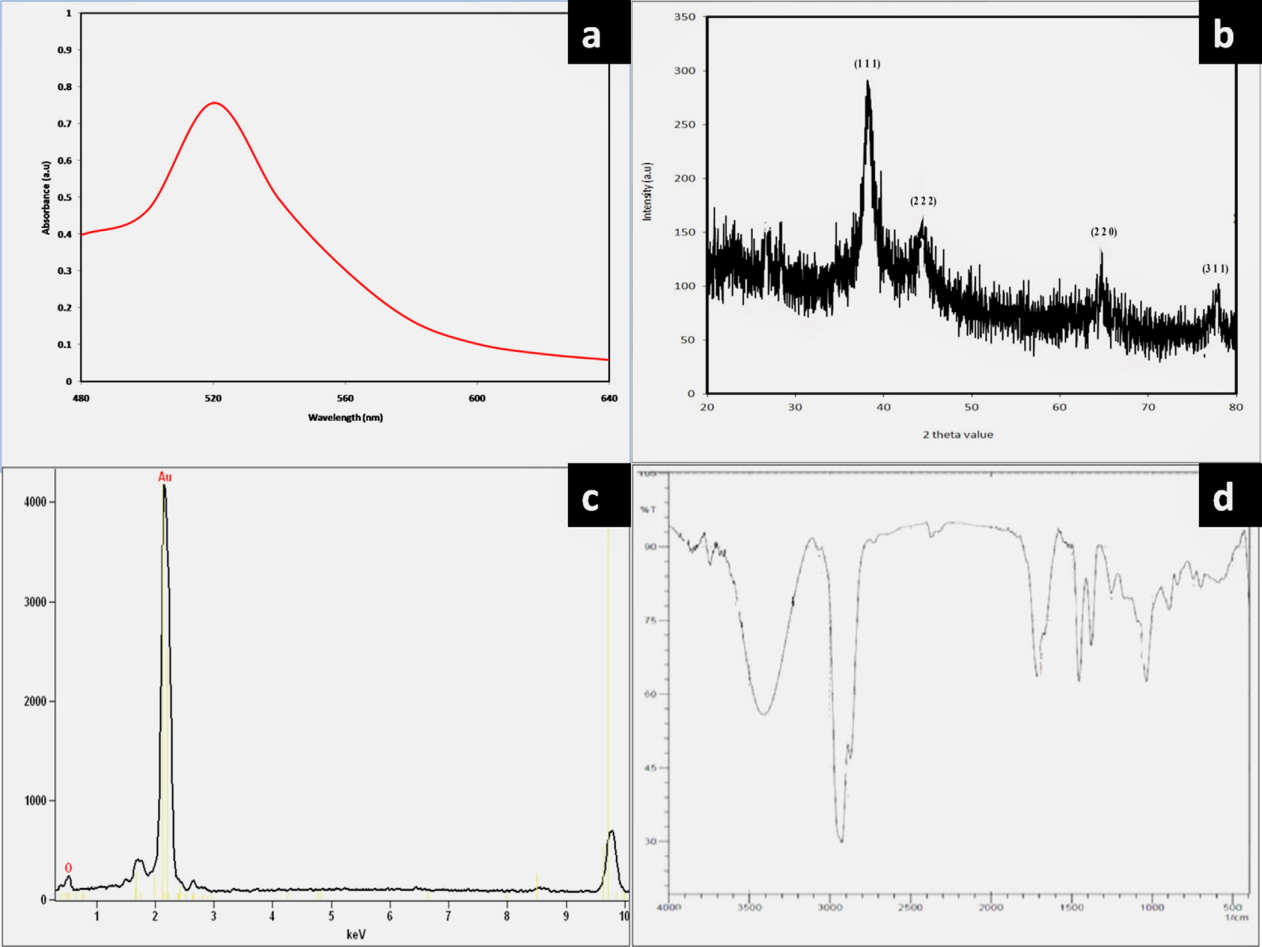
a) UV–vis spectrum of synthesized gold nanoparticles *Alternanthera bettzickiana*; b) XRD spectrum green synthesized gold nanoparticles; c) EDAX spectrum shows presence elemental gold; d) FTIR spectrum of green synthesized gold nanoparticles.

The formation of Au NPs in the final reaction mixture was further confirmed by UV–vis absorbance measurement which shows surface Plasmon resonance band (SPR). The result of UV–vis spectra (Fig. 2a) it clearly indicates that the sharp localized surface plasmon resonance band at 520 nm which is characteristic peak for nanogold [33,34]. UV–vis spectra the color change manifested is caused by SPR of the forming Au NPs. A characteristic sharp peak was observed at 530 nm which is a characteristic peak of gold. This single narrow peak indicates the formation of monodispersed Au NPs [29]. Particularly this peak revealed spherical shape of nanoparticles [35], which was confirmed by TEM image. This strong resonance is arises due to the excitation of surface plasmon vibrations of synthesized Au NPs. Similarly, reported that SPR band at 530 nm for Au NPs [36].

#### 3.2.2 X-ray diffraction analysis (XRD)

The XRD spectrum is used to identify the crystalline structure of biogenic synthesized Au NPs. The XRD pattern shown in (Fig. 2b) indicated production of Au NPs recorded at diffracted intensities ranges from 20 to 80° of 2θ angles. The reduced Au NPs have five different diffraction peaks at 2θ values of 38°, 44°, 68° and 77° attributed to the planes of (111), (200), (220) and (311), respectively. This result indicates that synthesized nanoparticles are composed of crystalline gold. Some of the minor peaks are presented due to the presence of organic moieties or biomolecules derived from plant extract [37].

#### 3.2.3 Energy dispersive X-ray spectroscopy (EDX) analysis

Elemental composition of Au NPs was characterized by EDAX. It shows that a strong signal was observed indicates that pure and crystalline nature of Au NPs. Detection of weak signal from Cl and Ag for chlorine and silver was also recorded (Fig. 2c) (Table 1).

**Table 1:** Elemental composition of green synthesized gold nanoparticles.

Element	Weight%	Atomic%
Cl K	6.00	19.53
Au M	52.35	56.05
Au L	41.65	24.42
**Total**	**100.00**	**100.00**

#### 3.2.4 Fourier transform infra-red spectroscopy

FTIR spectrum of biogenic synthesized Au NPs shows availability functional groups. More number of functional groups was present in Au NPs (Fig. 2d). The broad peak was observed at 3412cm^−1^ corresponds to O—H stretch, H–bonded of alcohols and phenols. A long narrow band was seen at the wave number of 2927 cm^−1^ indicates the presence of C—H stretched alkane groups. Weak bands were noted at 1758 and 1454 ^−1^ represents the C—C stretch (in-ring) of aromatics and N–O symmetric stretch nitro groups, respectively. Very weak bands were formed at 1250 and 1037 cm^−1^ corresponds to C–N stretched aromatic amines and aliphatic amines, respectively (Table 2). Similar results were reported that wave numbers signal stretching and vibrational bending of the peaks may be derived from phytoconstituents such as flavonoids, terpenoids, alkaloids and soluble proteins present in plants extracts and these may be responsible for the stabilization and reducing of Au NPs. This method is a rapid, effective, convenient environmental benign method. *A.bettzickiana* plant extract was successfully used for the fabrication of Au NPs. The plant extract was acted as both reducing and stabilizing agent in the conversion of gold ions to Au NPs. The results are similar to many studies reported in the literature [4,5,9, [38], [39]].

**Table 2:** FTIR spectrum of green synthesized gold nanoparticles.

Wave number (cm^−1^)	Bond	Functional groups
3412	O—H stretch, H—bonded	alcohols, phenols
2927	C—H stretch	Alkanes
1758	C=O stretch	carbonyls (general)
1454	C—C stretch (in–ring)	Aromatics
1327	N—O symmetric stretch	nitro compounds
1250	C—N stretch	aromatic amines
1037	C—N stretch	aliphatic amines
		

#### 3.2.5 Scanning electron microscopy analysis

**Fig. 3:**
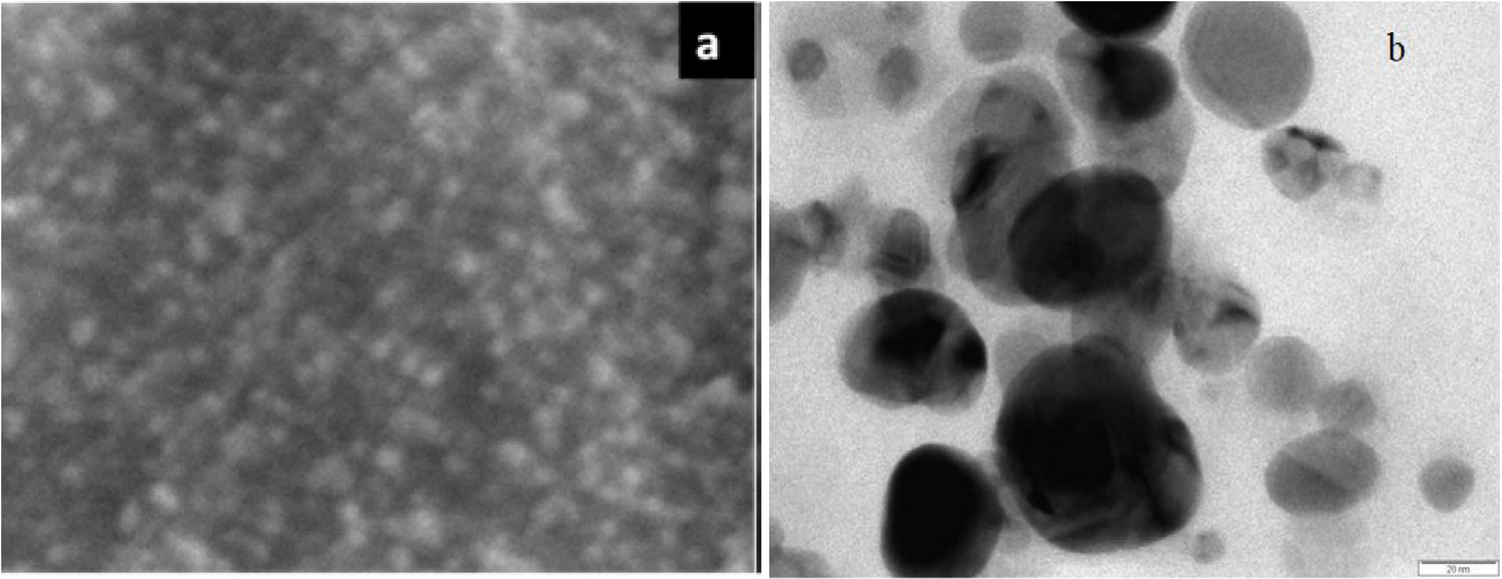
a) SEM image of gold nanoparticles; b) TEM image of bioreduced green synthesized gold nanoparticles.

From the scanning electron microscope (SEM) images it can be observed that predominantly spherical shaped particles of gold with clumps of aggregation. The size range of biogenic synthesized Au NPs found to be 80–120 nm at the scale bar of 200 nm **(**Fig. 3a).

#### 3.2.6 Transmission electron microscope

Size and morphological structure of reduced biogenic synthesized Au NPs was analyzed by TEM. TEM result shown in (Fig. 3b), it confirmed that the synthesized nanoparticles were in the nano range and of spherical shape. The size of the spherical shaped Au NPs ranges from 60 to 80 nm with uniform distribution viewed at 1000 nm scale bar.

#### 3.2.7 Zeta potential

Zeta potential (ZP) values reveal information regarding the surface charge and stability of the synthesized Au NPs. At different quantity of the *A*. *bettzickiana* leaves extract, there was little variation in the zeta potential value of Au NPs. However, Au NPs demonstrate lower ZP value at lower concentration of the extract, whereas higher values were obtained at higher concentration of the extract. The results of zeta potential value for Au NPs obtained from *A*. *bettzickiana* leaves extract were found to be −41.4, indicating the stability of the synthesized Au NPs.

### 3.3 Antibacterial activity

**Fig. 4:**
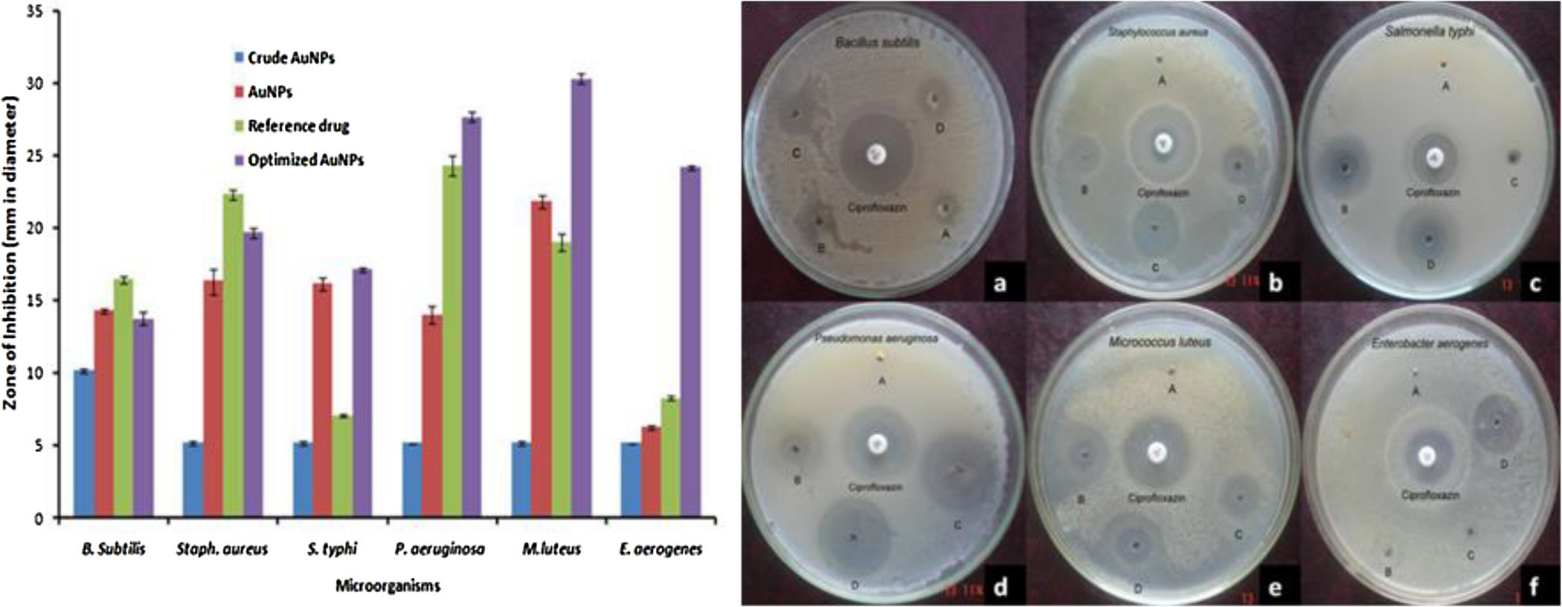
Antibacterial activity of green synthesized Au NPS against a) *B. subtilis*; b) *S. aureus*; c) *Salmonella typhi*; d) *P. aeruginosa*; e) *M. luteus*; f) *E. aerogenes.*

The present study revealed that Au NPs synthesized using aqueous leaf extract of *A*. *bettzickiana* showed has antibacterial property. The antibacterial activity of synthesized Au NPs was performed against *B. subtilis*, *S. aureus*, *S. typhi*, *P. aeroginosa*, *M. luteus*, and *E. aerogenes* by agar well diffusion method. Antibacterial activity of different types of Au NPs i.e. crude Au NPs, Au NPs, optimized Au NPs was compared with reference drug. The zone of inhibition (mm) of crude Au NPs, Au NPs, and optimized Au NPs against *B. subtilis* was found to be 10 ± 0.17, 14 ± 0.15, 16 ± 0.23 and 14 ± 0.43, respectively. Among the three types of Au NPs, optimized Au NPs shows maximum zone of inhibition. Zone formation against *S. aureus* was noted for optimized Au NPs found to be 19 ± 0.33 mm in diameter around the well. There is no zone formation was noted for crude Au NPs against a multidrug-resistant *S. aureus*. Likewise, crude Au NPs does not shown inhibition zone against *S. typhi*, *P. aeruginosa*, *M. luteus* and *E. aerogenes*. The Au NPs show the inhibition activity is 16 ± 0.88, 16 ± 0.44, 14 ± 0.58, and 22 ± 0.44 mm in diameter against *S. typhi*, *P. aeruginosa*, *M. luteus* and *E. aerogenes*, respectively. Maximum zone of inhibition was observed by optimized Au NPs against *M. luteus, P. aeruginosa* and *E. aerogenes* was found to be as 30 ± 0.33 mm, 28 ± 0.33 mm and 24 ± 0.17 mm, respectively which compared with other antibacterial agents (Fig. 4) (Table 3). Green synthesis of Au NPs (Au NPs) revealed the most significant bacterial effect and thus it could be used as a (potential) bacterial pathogen for various medical applications. The synthesized Au NPs have shown enhanced antibacterial activity [[38], [39], [40]]. It is very interesting that all these biogenic synthesized Au NPs show efficient antibacterial activity against certain bacterial strains, especially compared to chemically synthesized Au NPs which showed nearly no antimicrobial activity against similar strains [[41]]. The antibacterial activity may be due to the synergistic effect of the combination of Au NPs and extracts [42]. Au NPs are synthesized using diverse plant extracts have been used for investigating their antimicrobial activities against different microbes.

**Table 3:** Zone of inhibition against pathogenic microorganisms.

Zone of inhibition (mm in diameter)				
	Crude AuNPs	AuNPs	Reference drug	Optimized AuNPs
*B. Subtilis*	10 ± 0.17	14 ± 0.15	16 ± 0.23	14 ± 0.43
*S. aureus*	05 ± 0.17	16 ± 0.88	22 ± 0.33	19 ± 0.33
*S. typhi*	05 ± 0.17	16 ± 0.44	07 ± 0.10	17 ± 0.13
*P. aeruginosa*	05 ± 0.00	14 ± 0.58	24 ± 0.67	28 ± 0.33
*M.luteus*	05 ± 0.17	22 ± 0.44	19 ± 0.58	30 ± 0.33
*E. aerogenes*	05 ± 0.00	06 ± 0.15	08 ± 0.15	24 ± 0.17

### 3.4 Minimum inhibition concentration (MIC)

**Fig. 5:**
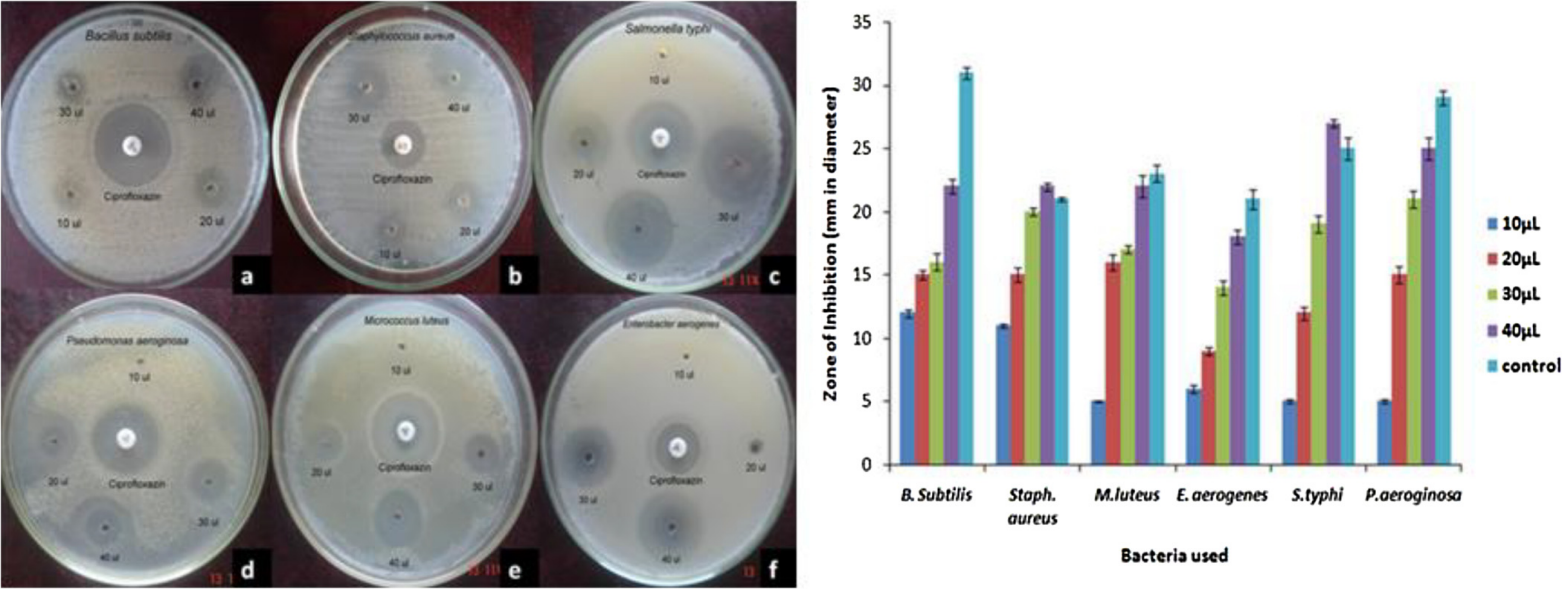
Minimum Inhibitory Concentration (MIC) against a) B. subtilis; b) *S. aureus*; c) *Salmonella typhi*; d) *P. aeruginosa*; e) *M. luteus*; f) *E. aerogenes.*

Minimum inhibition concentration (MIC) of synthesized Au NPs was determined against *Bacillus subtilis*, *S. aureus, S. typhi, P. aeruginosa, M. luteus* and *E. aerogenes* by agar well diffusion method. The concentration of Au NPs was varied from 10 to 40 μL. Zone of inhibition was increased as increasing the concentration of Au NPs against bacterial pathogens. The zone of inhibition in diameter was tabulated for the determination of minimum inhibition concentration (MIC). The clear zone formation starting at the concentration is considered as minimum inhibition concentration. The minimum concentration required to inhibit the growth of bacterial strains are 10 μL, 20 μL, 30 μL, and 40 μL against *B. subtilis, S. aureus, M. luteus, E. aerogenes, S. typhi* and *P. aeroginosa,* respectively (Fig. 5, Table 4).

**Table 4:** Minimum inhibitory concentration of gold nanoparticles against bacterial pathogens.

Zone of inhibition (mm in diameter)					
Bacteria used	10μL	20μL	30μL	40μL	control
*B. Subtilis*	12 ± 0.29	15 ± 0.33	16 ± 0.67	22 ± 0.58	31 ± 0.44
*S. aureus*	11 ± 0.17	15 ± 0.58	20 ± 0.33	22 ± 0.29	21 ± 0.17
*M.luteus*	05 ± 0.06	16 ± 0.58	17 ± 0.33	22 ± 0.88	23 ± 0.67
*E. aerogenes*	06 ± 0.29	09 ± 0.33	14 ± 0.58	18 ± 0.58	21 ± 0.73
*S.typhi*	05 ± 0.17	12 ± 0.50	19 ± 0.67	27 ± 0.33	25 ± 0.83
*P.aeroginosa*	05 ± 0.15	15 ± 0.67	21 ± 0.67	29 ± 0.88	29 ± 0.58

### 3.5 Anticancer activity of A. bettzickiana in human lung cancer cell line (A549) and its mechanism of action

**Fig. 6:**
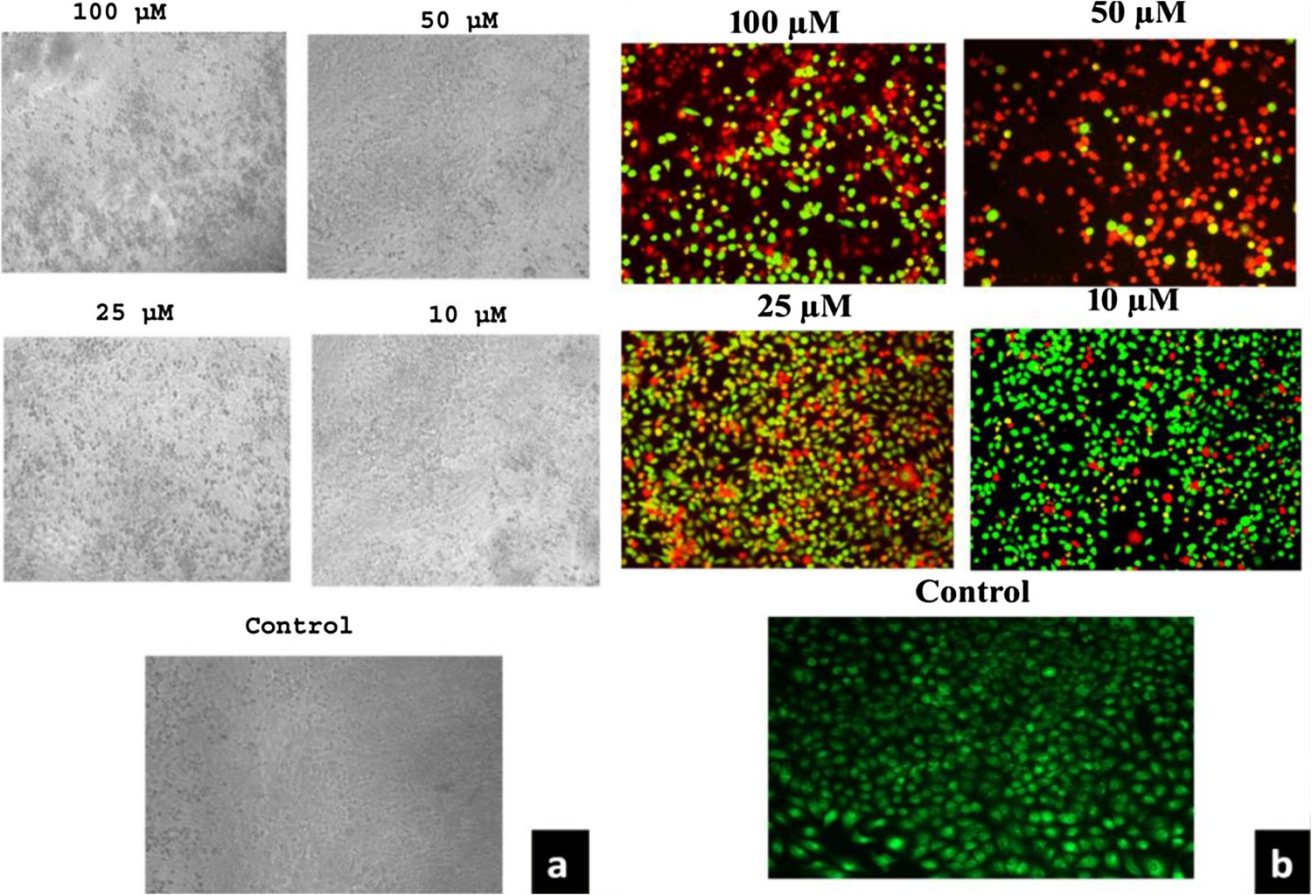
(a) Gold nanoparticles reduces the cell viability; b) AO/EtBr is used in evaluating the nuclear morphology of apoptotic cells.

Lung cancer cell line (A549) incubated with different concentrations of Au NPs synthesized by using *A. bettzickiana* leaf extract. The occurrences of morphological changes and cell viability was detected under the phase contrast microscope are represented in (Fig. 6(a)). There was no changes observed in the morphology of the cells, whereas nanoparticles treated cells showed irregular shaped, abnormal, cytoplasmic vacuolation and enlarged cells at the concentration of 50–100 μM of Au NPs. At a concentration from 10 to 20 μM of Au NPs treated cells does not shows any morphological changes. These changes occur due to the Au NPs induced stress showing significant morphological changes when observed under phase contrast microscope.

### 3.6 Lung cell line culture

**Fig. 7:**
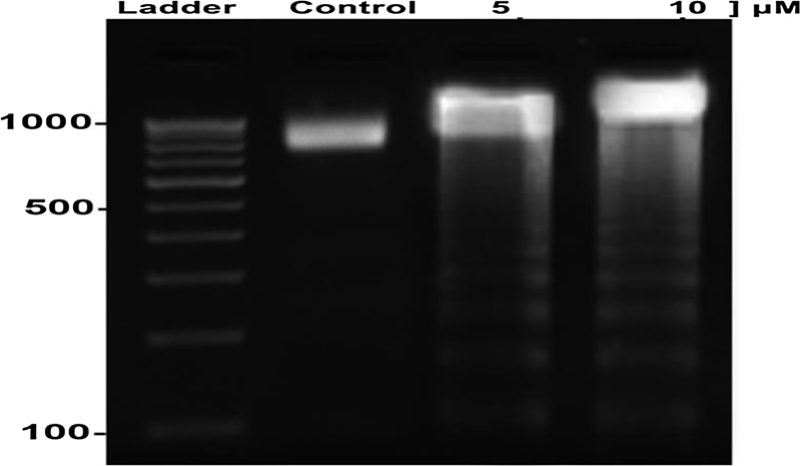
Effects of agarose gel showing the effect on internucleosomal DNA fragmentation in A549 cells.

The lung cancer lines were treated with Au NPs for 24 h and stained for nuclear morphological changes. Acridine Orange/Ethidium Bromide (AO/EtBr) was used for evaluating the nuclear morphology of apoptotic cells and visualized under fluorescent microscopy. Acridine orange is a vital dye which could be used on both live and dead cells, whereas Ethidium Bromide was used for staining only those cells that have lost their membrane integrity. The stained control cells showed nuclei having round and green colored nuclei. However, early apoptotic cells had fragmented DNA and stained as green colored nuclei. So that, green color stained cells represent viable cells, whereas reddish or orange represents late apoptotic cells. In the control, uniformly green live cells were observed. The result indicates Au NPs synthesized induce apoptotic death in human lung cell line cells (A549). Au NPs treated A549 cells showed more nuclear morphological changes compared to the control cells. After treatment, DNA was isolated run on agarose gel and visualized. Agarose gel showed DNA fragmentation in human lung cell line A549 cells (Fig. 6(b)). Au NPs treated A549 cells showed more nuclear morphological changes compared to the control cells. After treatment, DNA was isolated run on agarose gel and visualized. Agarose gel showed DNA fragmentation in human lung cell line A549 cells (Fig. 7).

### 3.7 Assay of effect of drug on apoptosis signaling pathway

**Fig. 8:**
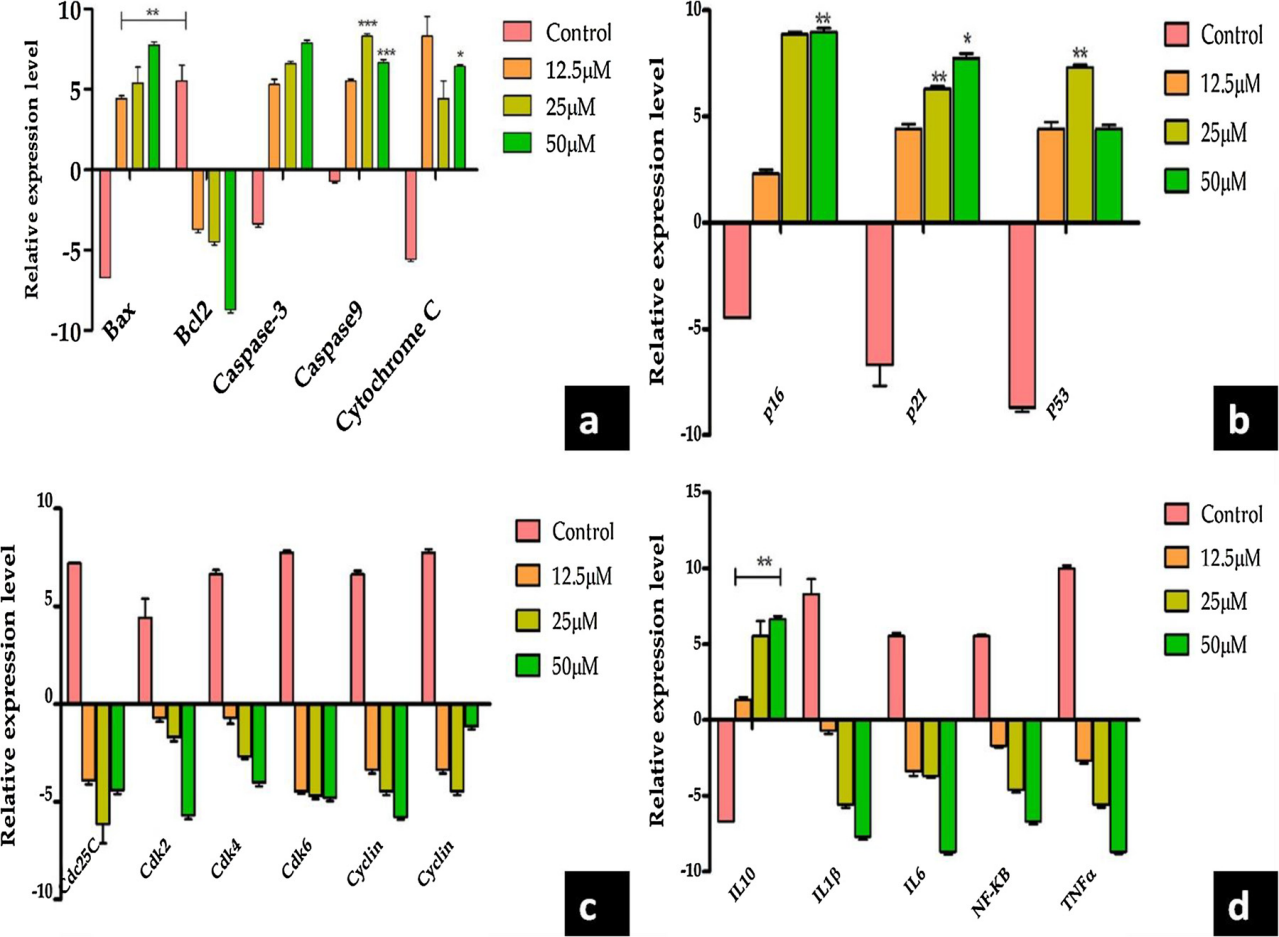
a) Immunoblotting analysis of protein expression; b) Effect of compound on tumor suppressor signaling pathway; c) Immunoblotting analysis of protein expression on cyclin cell arrest; d) Effect of drugs on the expression of inflammatory markers in A549 cells. (Experiments were performed in triplicates. The results are expressed as mean ± SE. Significant difference between treated and control group given as *(p < 0.05), ** (p < 0.01) and *** (p < 0.001).

Effect of drug on Apoptosis signaling pathway, Cells were treated with IC 50 Concentration of drug and RT-PCR analysis was performed for target genes. The band density of RT-PCR representing the target gene expression level and normalized against GAPDH. Values were expressed in terms of Mean ± SEM (n = 3). The lung cancer cell lines (A549) were treated with different concentration of green synthesized Au NPs which induces morphological changes in cells with the initiation of apoptosis. As a result, the caspase activity of A549 cells was examined shown figure. It clearly confirmed that treatment of cancer lines with different concentrations Au NPs caused a significant increase in caspase activation in cell lines. The concentrations of Au NPs 25 and 50 M were caused more significant (p < 0.001) increase in caspase -9 activation in cell lines. At the concentration of 50 M of Au NPs had the significant (p < 0.05) cytochrome C activation. Bax and BCl_2_ have average significant (p < 0.01) cell activation ability (Fig. 8(a)). Finally, this result concluded that Au NPs were significantly more active in A549 cells compared to control treatments. The mechanistic pathways of nanoparticles caused cellular damage and cell death still not yet fully understood. Au NPs treated cells were not viable, floated and roundedness. Some of the studies reported that cytotoxic effect was dependent on their size and shape of Au NPs [43,44]. Patra et al. [45] studied that A549 cells showed decrease in percentage of viability in the Au NPsconcentration range 0–30 nM with the average size of the nanoparticle to be 33 nm. Likewise [46], reported that apoptosis was induced by Au NPs in MCF-7 breast cancer cell lines via p53, 2012 caspase and Bax/Bcl-2 pathways [47]. IH441 cell lines and 3T3-L1 cell lines, respectively.

### 3.8 Effect of drug on tumor suppressor signaling pathway

**Fig. 9:**
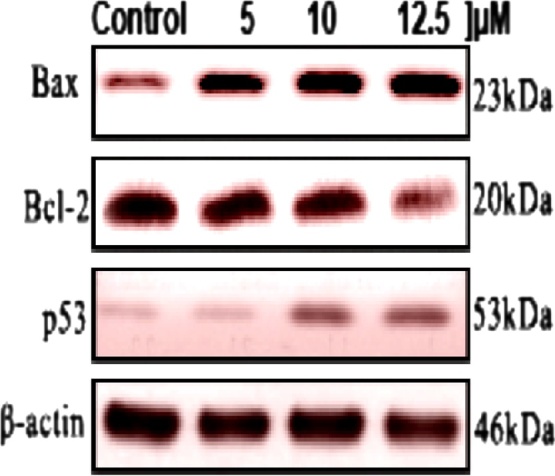
Immunoblotting of gene expression.

**Fig. 10:**
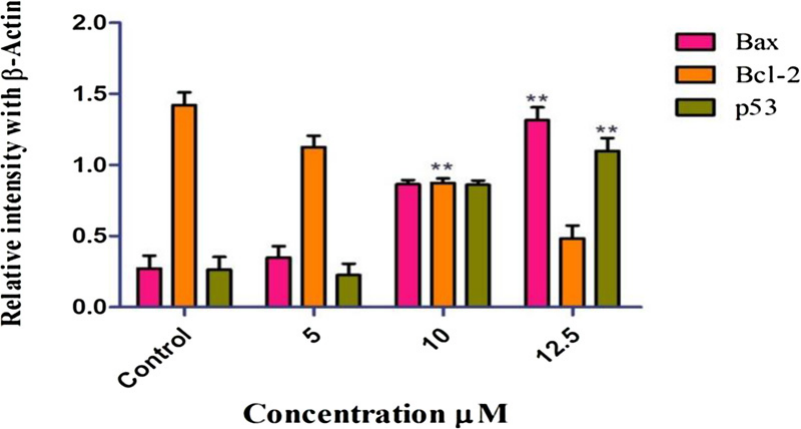
Effect of gold nanoparticles on gene expression in Bax, Bcl-2 and p53. (Experiments were performed in triplicates. The results are expressed as mean ± SE. Significant difference between treated and control group given as *(p < 0.05), ** (p < 0.01) and *** (p < 0.001).

The band density of RT-PCR representing the target gene expression level and normalized against GAPDH. Values were expressed in terms of Mean ± SEM (n = 3). Comparisons were done by Tukey`s comparison test. *** −p < 0.001, ** −p < 0.01 and *−p < 0.05 compared to control group (Fig. 8(b)). Western-blot analysis performed on human lung cancer (A549) cell line revealed decreased CDC2, CDK2, CDK4, and reduction of the proteins cyclin B. The expression levels of Survivin, COX-1, COX-2, PGE2 were significantly decreased after treating with plant-based silver nanoparticles in A549 human lung cancer cell lines (Fig. 8(c)). After the treatment of cancer cell lines with different concentration of Au NPs, the expression of dc25C, CDK2, CDK4, CDK6, and cyclin A were significantly reduced. The lung cancer cell lines were exposure to green synthesized Au NPs may change the gene or protein expression of the cells. This exposure could be result in a significant up-regulation of the pro-inflammatory genes like IL0, IL1β, IL-6, NF—KB, and TNF-α. (Fig. 8(d)). A549 cells were treated with indicated concentration of drugs for 24 h. The control group was treated with 0.1% DMSO for 24 h. Cell lysate was prepared and protein level was determined by Western blotting analysis. Equal amounts of total proteins (40 μg) were subjected to 12% SDS–PAGE gels and transferred onto PVDF membranes. The membranes were then probed with the indicated antibodies, and the proteins were visualized using an ECL detection system. β- Actin was used as an internal control. The expression levels were quantified using the Image *J* analysis. The data are representative of three independent experiments. **P < 0.05, *P < 0.01 and compared with the respective control group. The experimental results are expressed as the mean ± SEM and are accompanied by the number of observations. A one-way analysis of variance (ANOVA) was performed using the Graph Pad Prism 5.0 software. Two-tailed unpaired Student’s *t*-test was used for statistical analysis of the data. The effects of Au NPs on apoptosis-associated genes was analyzed in this study. When the lung cancer cell lines were treated with different concentration of Au NPs, the level of Bax, Bcl-2 and p53 mRNA in cancer cell lines was significantly (p < 0.01) up-regulated (Fig. 9, Fig. 10).

### 3.9 Heat map summary and hierarchical clustering of gene expression

**Fig. 11:**
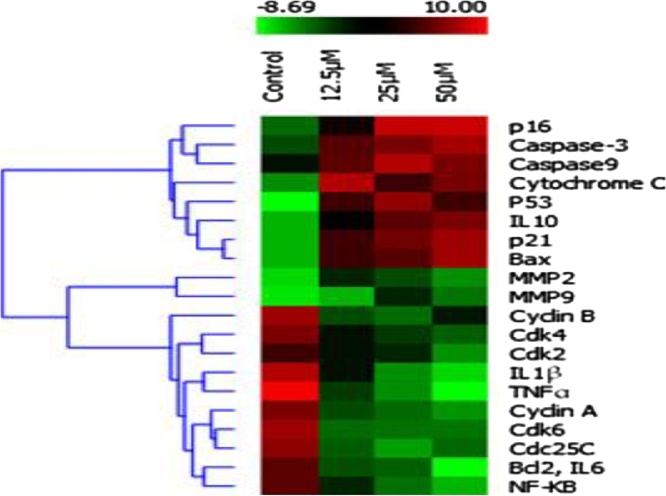
Heat map summary and hierarchical clustering of gene expression.

Gene expression data were obtained using RT-PCR analysis. The cluster diagram represents expressed genes with p < 0.05 and Delta > 1.8. Each column represents a single gene and each row represents different treatment groups. Expression levels are colored green for low intensities and red for high intensities (see scale at the top). The cluster is color coded using red for upregulation, green for down regulation, and black for median expression (Fig. 11).

### 3.10 In-vitro toxicity studies of green synthesis of Au NPs using zebra fish embryo model

**Fig. 12:**
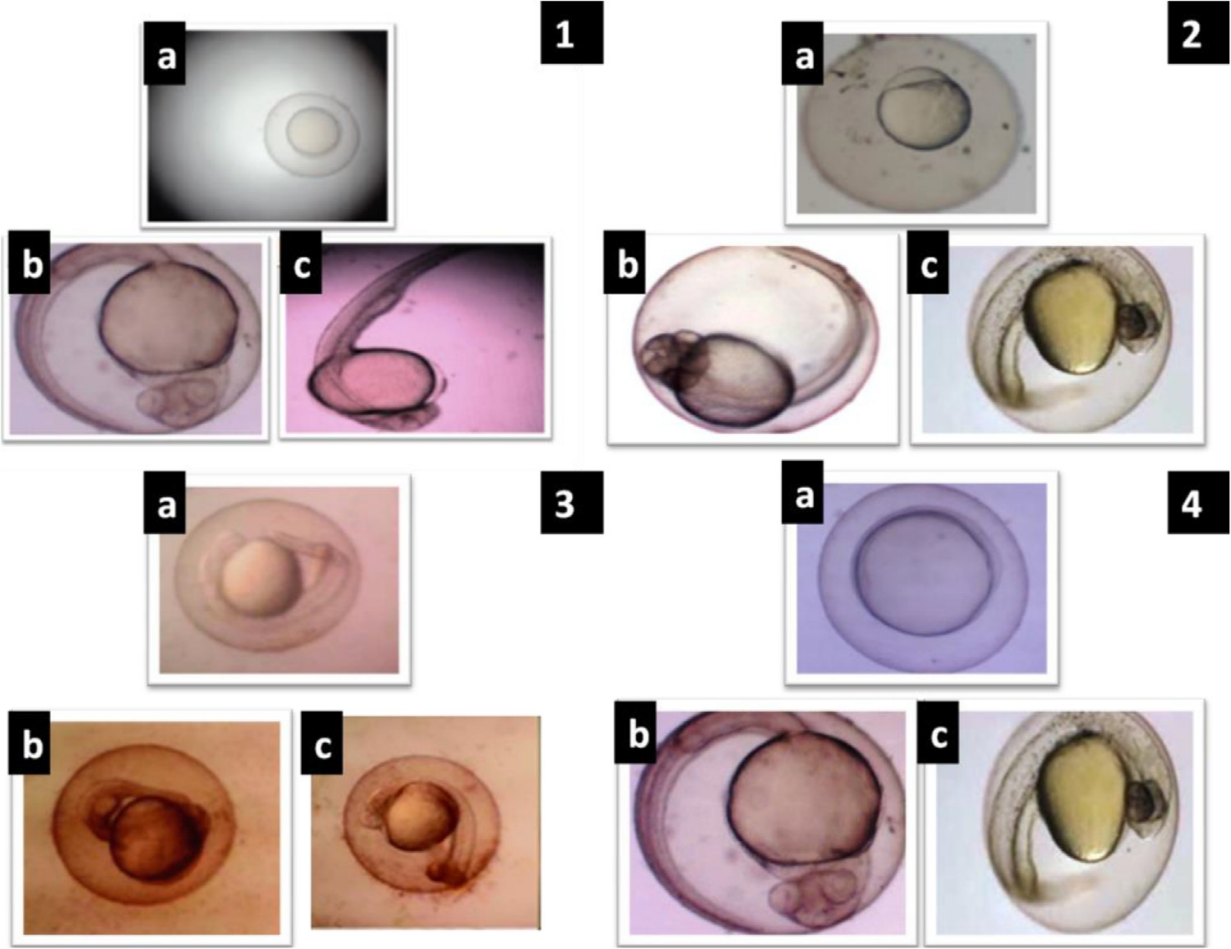
1. a) Normal development of zebra fish embryo at different time interval (A-12 h, B-36 h and 48 h); 2. b) Normal development of zebra fish embryo treated with plant based gold nanoparticles concentrations of 12μM; 3. c) Normal development of zebra fish embryo treated with plant based gold nanoparticles concentrations of 25μM; 4 d) Normal development of zebra fish embryo treated with plant based gold nanoparticles concentrations of 50 μM.

To evaluate the possibility toxicity of zebra fish (*Danio rerio*) embryos exposure to different concentrations (12 μM, 25 μM and 50 μM) of biogenic synthesized Au NPs by measuring mortality and hatching rate. The developmental stage of zebra fish was observed at different time interval like 12, 36 and 48 h. The development stage of embryo of nanoparticles treated zebra fish was compared with the normal development embryos (Fig. 12–1(a, b, and c)). In normal development stage embryo and tail was developed at 36 h and 48 h respectively. So that, the hatching period of normal embryos from 36 to 48 h. As shown in Fig. 12–2(a, b and c), the lower concentration (caused slight significant difference. Fig. 12–3(a, b and c) showed low inhibition of hatching rate after exposing embryos to Au NPs at the concentration of 25 μm. Fig. 12–4(a, b and c) showed strong inhibition rate of hatching after the treatment with 50 μm concentrations of Au NPs. At 48 h of time, the tail formation in zebra fish embryo was much affected than the control. Moreover, large amounts of dark material were found in the gut tract of fish embryo but not in the control. No toxicity was observed in the biogenic synthesized Au NPs to the concentration of 25 μM while fed the Danio rerio (Zebra fish) (Fig. 12).

## 4 Conclusion

In this work, we describe a simple, quick and reproducible method for the environmentally friendly synthesis of Au NPs without the need for expensive reducing agents. Gold ions were biologically reduced to NPs by *A.bettzickiana* leaf extracts. The biogenic synthesized Au NPs is characterized using TEM, SEM, EDX, XRD, FTIR, and UV–vis spectroscopy. The Au NPs reported to exhibit potent anti bacterial activity. The biogenic synthesized Au NPs initiate the cancer cell death by lessening cell proliferation, alteration in mitochondrial membrane potential, DNA fragmentation and apoptosis in Lung cancer cell line. Au NPs affect entrance of cellular M phase. It has been proposed that Au + might induce P53 and other cell cycle genes thus preventing cells from entering M phase and promoting apoptosis. Studies on the normal embryonic development of *Danio rerio* (zebrafish) are important not only to amplify the knowledge about the developmental process but also to understand the time specific developmental process in course of particular fish species. In view of the above we conclude that the biogenic synthesized Au NPs can be used for various biological purposes, particularly, in cancer research. This opens the possibility for the use of Au NPs for drug delivery, oral or intranasal, without interfering with the human microbiota.

## Conflict of interest

The authors declare that there is no Conflict of interest between them. I warrant that the article is the Authors' original work. I warrant that the article has not received prior publication and is not under consideration for publication elsewhere. On behalf of all Co-Authors, the corresponding Author shall bear full responsibility for the submission.
